# A Case Study in Personal Identification and Social Determinants of Health: Unregistered Births among Indigenous People in Northern Ontario

**DOI:** 10.3390/ijerph16040567

**Published:** 2019-02-16

**Authors:** Chris Sanders, Kristin Burnett

**Affiliations:** 1 Department of Sociology, Lakehead University, 955 Oliver Road, Thunder Bay, ON P7B 5E1, Canada; 2 Indigenous Studies, Lakehead University, 955 Oliver Road, Thunder Bay, ON P7B 5E1, Canada; kburnett@lakeheadu.ca

**Keywords:** Canada, Indigenous people, geographic disparity, personal identification, birth registration, social determinants

## Abstract

Under international law, birth registration is considered a human right because it determines access to important legal protections as well as essential services and social supports across the lifespan. Difficulties related to birth registration and the acquisition of personal identification (PID) are largely regarded as problems specific to low-income countries. For Indigenous people in northern and rural Canada, however, lack of PID, like birth certificates, is a common problem that is rooted in the geography of the region as well as historical and contemporary settler colonial policies. This communication elucidates the complicated terrain of unregistered births for those people living in northern Ontario in order to generate discussion about how the social determinants of health for Indigenous people in Canada are affected by PID. Drawing on intake surveys, qualitative interviews and participant observation field notes, we use the case study of “Susan” as an entry point to share insights into the “intergenerational problem” of unregistered births in the region. Susan’s case speaks to how unregistered births and lack of PID disproportionately impacts the health and well-being of Indigenous people and communities in northern Ontario. The implications and the need for further research on this problem in Canada are discussed.

## 1. Introduction

Birth registration is considered a fundamental human right and one that “continues to be overlooked” with regard to its significance as a social determinant of health [1]. According to the United Nations “[a]ccessibility [to birth registration centres] is influenced by location and terrain, infrastructure and the availability of transportation. The greater the distance to the registration centre, the higher the financial and opportunity costs for the family” [2,3]. It should be noted that “[w]hile birth registration does not of itself guarantee education, health, protection and participation in society, its absence can put these fundamental rights beyond the reach of those already on the margins of society” [1]. Research on low income countries has also revealed that there are clear links between health outcomes and birth registration [4,5,6,7,8]. Indeed, lack of a birth certificate has cascading consequences on an individual’s ability to access essential services and social supports across the lifespan. For instance, in Canada individuals without a birth certificate cannot prove their age and, as a result, are disqualified from receiving child benefits or old age security [9].

Difficulties related to birth registration and the acquisition of personal identification (PID) are largely regarded as a problem associated with low-income countries with poor civil registration systems [2,9,10,11,12]. However, in Canada there continue to be groups that face significant barriers to accessing essential social and health services because they do not possess certain forms of PID like birth certificates [13]. In rural northern Canada, in particular, lack of PID disproportionately affects Indigenous people and is rooted in both the geography of the region but also in historical and contemporary settler colonial policies.

In Canada, a birth certificate is the foundational piece of PID that enables access to most government services and supports like child and family benefits, social housing, social service supports (e.g., social assistance, Old Age Security and Employment Insurance or EI), emergency food supports (e.g., food banks) and so on. As well, individuals without a birth certificate cannot open bank accounts, which in turn limits one’s ability to receive electronic payments, make significant purchases or establish and build credit. Indeed, the possession or lack thereof, of a birth certificate has cascading and cumulative effects on an individual’s well-being that are all connected with Social Determinants of Health (SDoH) [14,15]. When combined with settler colonialism and geography, the consequences of no-PID become more pronounced. Lack of PID for many individuals poses an almost insurmountable barrier and the administrative and financial requirements imposed by the provincial and the federal governments to obtaining PID only make matters worse.

According to the World Health Organization, SDoH are “the environmental conditions in which people are born, grow, live, work and age—conditions that together provide the freedom people need to live lives they value” [16]. For instance, we know that individuals and communities living without access to potable water, affordable and nutritious food, safe housing, financial support and health care, have disproportionately poorer health outcomes [17]. While most G7 countries have social support systems in place that attempt to address some of those basic social and material needs, for Indigenous people around the globe settler colonialism and racism generates additional barriers to access [18].

For Indigenous people in Canada, settler colonialism and the dispossession of land (historical and ongoing) have been identified as the most significant of the Social Determinants of Health (SDoH) [19,20,21]. Indeed, we cannot have an informed and productive discussion about the health and well-being of Indigenous people and their communities without considering their relationships to land and the impact that settler colonialism has had on those relationships [22]. Dislocation from land, culture and resources, lower educational attainment rates, high rates of food insecurity and limited access to health care, all of which are compounded by racism, have ensured that Indigenous peoples experience disproportionately poorer health outcomes than their non-Indigenous counterparts [23].

This short communication is an effort to elucidate the complicated terrain of unregistered births and lack of PID for those people living in rural northern Ontario, and is intended to generate discussion about how the social determinants of health for Indigenous people in Canada are affected by PID. We use an illustrative case study as an entry point to share insights that have been generated from a community-based research project, in which we are evaluating the need and effectiveness of an ID services program for the region.

## 2. Materials and Methods

### 2.1. Setting

Northern Ontario constitutes about 87% of Ontario’s total landmass and is located within the Robinson Superior Treaty and Treaties 3, 5 and 9 [24], yet the region only contains about 780,000 residents (or approximately 6% of the province’s total population) [25]. Twenty percent of the population in northern Ontario is of Indigenous ancestry as compared to 2% for the rest of Ontario. There are 49 First Nation communities, 32 of which are only accessible by plane or briefly during the winter by seasonal ice roads [26]. The location of these communities makes accessing essential health and social services extremely challenging.

The size of northern Ontario, particularly the provincial far north, presents unique challenges in regard to health and social services delivery. In northern Ontario, Indigenous people transverse multiple spaces and boundaries when accessing health care services. First Nations residing in northern Ontario live on-reserve, off-reserve and frequently both. In other words, they may live in the city and regularly travel to their home community to visit family, participate in cultural activities and to pursue land and water based food procurement activities [27].

Given the geographic diversity and location of Indigenous peoples throughout the northern portion of Ontario it is impossible for us to definitively count the exact number of individuals whose births were not registered and subsequently do not possess PID. However, an assessment of the challenges and barriers faced by individuals acquiring PIDs and its impact on health outcomes first requires that we hold conversations with community members living in a range of locations: urban, road access reserves (both near and distant from cities and towns) and fly-in access reserves/First Nations. This initial phase of the project is detailed below.

### 2.2. Community Partner and Relationship

During the winter of 2018 we began working with the Kinna-aweya Legal Clinic to evaluate their “Awenen Niin (Who Am I?)” ID Services program. Awenen Niin had received funding through the Local Poverty Reduction Fund to sustain its ID service program. Located in Thunder Bay, the Kinna-aweya Legal Clinic provides “legal advice and assistance to residents of the District of Thunder Bay, particularly Aboriginal people, who need assistance with poverty law issues” [28]. The clinic has branch offices in Geraldton and Marathon, both small towns in northern Ontario with populations of less than 5000 residents. The Awenen Niin program began in 2012 by holding ID clinics to assist vulnerable clients with PID form completion, gathering required information and providing ongoing case management and referrals to appropriate community and governmental services and benefits. In 2015, these services expanded to include an “ID Bank” that securely stores PID and notarized copies of PID for clients who are susceptible to losing PID due to precarious housing, mental illness or other such factors. Since 2012, more than 1200 people have been helped through the Awenen Niin program.

### 2.3. Community-Based Design

Kinna-aweya identified assistance obtaining PID as a pressing and serious gap in service provision and the Awenen Niin Program fills this service vacuum that is not provided by any other agency in Thunder Bay and northern Ontario more broadly, including Service Ontario and Service Canada. Kinna-aweya personnel have specifically identified this problem as situated within a social determinants framework. Our research practice adheres to the principles outlined in “Ownership, Control, Access and Possession (OCAP) or Self-Determination Applied to Research” [29].

This is a community based research (CBR) project that is guided entirely by the needs of our community partner [29]. Our research design is flexible and adaptable. Drawing on the bus metaphor used by Loretta Jones in her 2006 keynote address to the Community of Campus Partnerships, we approach this process like a shared bus journey [30]. We do not begin our research with communities with a predetermined agenda; rather we encourage community members/partners to drive and shape the research process so that it suits their needs and desires and to return to the bus metaphor, results in a shared destination. As our research proceeds, we will adjust and reassess project objectives to meet the needs and interests of our community partner. Thus, to meet the objectives established by Kinna-aweya, our role within the project is: (1) to evaluate the breadth of the problem regarding lack of PID in the region especially as it disproportionately affects Indigenous people; (2) to create a profile of those individuals accessing the Awenen Niin program; (3) to identify and map in a systematic manner the challenges and barriers regarding the acquisition of PID; (4) to document ID services throughout the province and the populations that different ID services serve and how; and (5) to identify the ways in which various organisations are trying to address the problem.

It is worth noting that although this issue is largely invisible to non-Indigenous academics and researchers, it has been identified as a serious challenge for Indigenous people and communities for quite some time. It is for this reason that presenting these findings is particularly important and central to using our research for advocacy and raising awareness as part of the community-based research process.

### 2.4. Participants and Recruitment

Exploration of this issue began in February 2018 when the authors were part of a team invited to serve as independent evaluators for the Kinna-aweya Legal Clinic’s Awenen Niin ID Services program. Qualitative and quantitative data collection began in Thunder Bay, Ontario in June 2018 and is ongoing. Data consist of client ID clinic intake surveys (*N* = 116), one-on-one semi-structured interviews with Kinna-aweya personnel and other service providers in Thunder Bay working on ID services (*N* = 5), informal conversations with Kinna-aweya personnel and health care providers and community organization members (*N* = 10) and our close involvement with the Thunder Bay “ID Action Group,” which meets monthly to track the progress of local ID services and “ID clinics” and develop advocacy strategies around PID. We have attended and taken field notes at nine of these meetings (*N* = 9). The ID Action Group consists of health care and social services providers, academics, community organization members, representatives from Indigenous organizations and service providers and other interested stakeholders (for example, a person from the local library attends and has made municipal space available for ID clinics).

### 2.5. Interview Procedure

Semi-structured interviews were conducted by both authors with participants on a one-on-one basis. The interviews were recorded and transcribed verbatim.

### 2.6. Analysis

Though the project is ongoing, we use a case study approach [31] to analyse the story of one young woman and her family. While extreme, the case illustrates a range of challenges that the research has uncovered to date that we believe are unique to this region and Indigenous populations. Data comes from interview transcripts and our field notes taken during one-on-one interviews, informal conversations and meetings and monthly participation with the ID Action Group. Our analytic approach uses the case as a heuristic example [32] that enables us to inductively build upon a social determinants framework to understand the unregistered birth problem in northern Ontario.

### 2.7. Ethics Approval and Consent to Participate

Participants gave informed consent prior to taking part in the study; the consulting process included information about the researchers and purpose of the study. The protocol for this study received ethics approval from the Lakehead University REB #1466548.

## 3. The Case of “Susan”: An Intergenerational PID Problem

Informal estimates based upon early data collection suggest that there are high rates of unregistered births in fly-in First Nations for a variety of structural and social reasons. This poses particular challenges as funding for essential services on-reserve is based on a per capita funding formula. Indeed, participants have shared insights from their professional work about people leaving their communities in their forties never having been issued a birth certificate. Our research mapping process has determined that the birth certificate is the foundational piece of PID that enables people to obtain other key pieces of PID (e.g., provincial health card, social insurance number, driving license). Without PID people become severely limited in their ability to access health and social services, obtain employment and bank accounts, secure safe housing and enrol in schools to name but a few key services. Significantly, for Indigenous people, a birth certificate that includes parental information is necessary to be legally registered as an Indian under the Indian Act, which in turn enables access to additional health and social services.

To illustrate the scope of the problem of PID in the region and the implication it has for the social determinants of health, we draw on the case of “Susan Waboose” (a pseudonym), a young mother who came to the Kinna-aweya ID clinic for assistance with obtaining PID for her daughter who needed PID to enrol in school and to receive non-insured health benefits. We chose Susan’s case because it illuminates the “intergeneration problem”, which happens when multiple generations of one family either lack birth registration or require a replacement birth certificate that has been lost, incorrectly issued in the first place or was never received from the issuing institution. Details about Susan’s case come from our field notes and interview data. According to interviewees, while it was Susan who first initiated the case file at Kinna-aweya, the case quickly became about mother, daughter and grandmother:

“Well, Susan’s case is actually about an entire family. So, originally Susan, who is the mom, comes in with her mother, the grandma. Susan’s daughter had been in the grandma’s care since the child was born but we told them in advance that we needed the mom [Susan] to fill out the forms because she’s the parent who gave birth. So, the mother and grandmother are kind of a team and come in together, which itself was a challenge because they both had to take off time from work and take the bus to get here among other issues.

It turns out it’s a delayed birth registration situation because the daughter wasn’t registered at the time of birth. So, to register the little one’s birth, you also need mom’s [Susan] birth certificate because you have to include proof of mother’s maiden name when you do a delayed birth registration. Mom [Susan] doesn’t have her birth certificate anymore, so we have to apply for that first before we can begin to apply for the daughter’s delayed birth registration.

So, we apply for mom’s [Susan’s] replacement birth certificate and it’s taking a long time. So I call to make an inquiry and eventually we receive it only to find out that the mother’s [Susan] birth place was listed incorrectly on her birth certificate. Who knows how that happened but we’re finding it’s not an uncommon occurrence among our clients to have records with mistakes or inconsistent details. So now we need to apply to amend the place of birth on the mother’s [Susan] birth certificate, which means we now need the Grandma’s birth certificate. Grandma doesn’t have hers, either. So then we order grandma’s birth certificate, which we’re still waiting on.

This means we need to pay a fee to fix a mistake that God-knows-who made twenty years ago [on Susan’s birth certificate]. All of this just to get the daughter’s birth registered so she can attend school and receive benefits. Keep in mind this process takes a long time and costs a lot of money for the client and they’re not able to obtain services until the paperwork is fixed and completed.”

This case is ongoing and has yet to see resolution wherein the child would be issued her birth certificate so that she can obtain essential services and supports.

## 4. Discussion

The case of Susan begins to shed light on a number of issues underlying the difficulty of obtaining PID in northern Ontario, a problem that disproportionately impacts Indigenous people and, in turn, affects their access to health and social services. What is clear from the case of Susan and her family is that for people whose birth was never registered, obtaining delayed birth registration is a complicated, time-consuming and costly process. Yet without this primary record, getting access to health and social services, vaccines, child benefits, schooling and so forth is difficult if not impossible for already under-serviced people.

Susan’s case also illustrates that for many Indigenous people living in northern Ontario, this is an intergenerational problem that affects multiple members of a family and has long-term health consequences. There are many reasons that this has become an intergeneration problem for Indigenous people, ranging from the history of residential schools [33,34,35]; the “sixties scoop” wherein large numbers of Indigenous children were removed from their families and communities and adopted out to predominantly non-Indigenous families in Canada and the United States [36,37,38,39,40]; women who were (and continue to be) forced to leave their northern communities to give birth [41,42,43]; fear of increased state surveillance and violence; and the fact that PID is often seen as irrelevant in small communities where members know everyone and have lived on these territories since time immemorial. Further, challenges regarding literacy in English or French (the official languages of service in Canada) are exacerbated when it comes to birth registration for those women who are evacuated to more southern locations like Thunder Bay for childbirth. The Thunder Bay Regional Health Sciences Centre, like many hospitals across the province, does not have a policy of ensuring that new mothers register births before they are discharged. Leaving the hospital without registering a birth makes it more likely that barriers, such as prohibitive cost and limited access to the internet where many state services are increasingly applied for and provided [44], will result in a birth not being registered. This, in turn, sets into motion a series of cascading effects that ensure the child will not receive those social and health services associated with the possession of a birth certificate.

As a result of these structural barriers compounded by settler colonialism and geography, this young girl (Susan’s daughter) cannot access services available to most people living in Canada. Exacerbating the matter, the entire family has also not been accessing many essential supports and services for decades owing to the family members’ missing and invalid forms of PID. Significantly, we need to think about the cumulative effects that these decades have had on the health outcomes of each individual as well as the family unit.

Since we began using the ID clinic intake forms to generate a client profile in June 2018, 113 forms have been completed (detailing information on 139 clients as parents often apply for multiple children on a single form). Over half of the individuals seeking to obtain some form of PID or register a birth were women. More than 80 percent of these clients identified as Indigenous and 15.9 percent of the clients sought either a delayed birth registration or their first birth certificate. When we presented these data at the monthly ID Action Group, a representative from Service Canada expressed surprise and, indeed, was alarmed at how high the rates were for delayed birth registration and first birth certificate. Clearly this problem is more serious than anyone anticipated as well as unrealized to people outside of these communities.

Susan’s case is indicative of many of the stories and experiences that have been shared with us since we began working with Kinna-aweya. Notably, as a function of our mapping process we have come to understand that ID clinics in southern and urban regions of Ontario tend to be organized around replacement ID services (where the birth was registered), a process that is relatively easier and more expedient and less costly than applying for delayed birth registration. It is also uncommon for PID services in southern Ontario to encounter multi-generation families without birth certificates. Indeed, based on our conversations with select service providers in some southern cities of Ontario, they report that it is rarer for them to perform delayed birth registrations. Hence, the problem of PID in Ontario appears to be more pronounced and unique in the rural north and in Indigenous communities.

## 5. Conclusions

This case study illustrates that the challenge of obtaining PID is a problem even in G7 nations and that the failure to possess this piece of ID can have cascading effects that can be intimately linked to the social determinants of health. Overall, we argue that this is an issue that has gone largely unrecognized as it relates to larger issues of health and well-being and settler colonialism.

It is important to note that there are no good data on unregistered births (almost by definition) in Ontario. We have received reports from key informants that certain fly-in access First Nation communities have anywhere from 50–75% of children whose births are unregistered. It is difficult to document or even know the extent of this problem, though Kinna-aweya ID clinics have found many cases. Our work is underway to further explore the extent of unregistered birth among Indigenous people, the structural challenges they face in trying to obtain personal identification and the long-term health consequences of not having PID. It is also important to note that the problem of PID and unregistered births is likely not confined to Ontario or Canada more broadly but most likely affects other settler states with Indigenous populations like Australia or New Zealand.
